# Rh-CSF1 attenuates neuroinflammation via the CSF1R/PLCG2/PKCε pathway in a rat model of neonatal HIE

**DOI:** 10.1186/s12974-020-01862-w

**Published:** 2020-06-10

**Authors:** Xiao Hu, Shirong Li, Desislava Met Doycheva, Lei Huang, Cameron Lenahan, Rui Liu, Juan Huang, Shucai Xie, Jiping Tang, Gang Zuo, John H. Zhang

**Affiliations:** 1 Department of Neurology, Guizhou Provincial People’s Hospital, Guiyang, 550002 China; 2 Department of Physiology and Pharmacology, Loma Linda University, Risley Hall, Room 219, 11041 Campus Street, Loma Linda, CA 92350 USA; 3 Department of Neurosurgery, Loma Linda University, Loma Linda, CA 92350 USA; 4 Bvrrell College of Osteopathic Medicine, Las Cruces, NM 88003 USA; 5 Institute of Neuroscience, Chongqing Medical University, Chongqing, 400016 China; 6 Department of Hepatobiliary Surgery, Affiliated Haikou Hospital of Xiangya Medical College, Central South University, Haikou, 570208 Hainan China; 7 Department of Neurosurgery, Taicang Hospital Affiliated to Soochow University, Taicang, Suzhou, 215400 Jiangsu China; 8 Department of Anesthesiology, Loma Linda University, Loma Linda, CA 92350 USA

**Keywords:** Hypoxic-ischemic encephalopathy, CSF1, CSF1R, PLCG2, Neuroinflammation, Microglia

## Abstract

**Background:**

Hypoxic-ischemic encephalopathy (HIE) is a life-threatening cerebrovascular disease. Neuroinflammation plays an important role in the pathogenesis of HIE, in which microglia are key cellular mediators in the regulation of neuroinflammatory processes. Colony-stimulating factor 1 (CSF1), a specific endogenous ligand of CSF1 receptor (CSF1R), is crucial in microglial growth, differentiation, and proliferation. Recent studies showed that the activation of CSF1R with CSF1 exerted anti-inflammatory effects in a variety of nervous system diseases. This study aimed to investigate the anti-inflammatory effects of recombinant human CSF1 (rh-CSF1) and the underlying mechanisms in a rat model of HIE.

**Methods:**

A total of 202 10-day old Sprague Dawley rat pups were used. HI was induced by the right common carotid artery ligation with subsequent exposure of 2.5-h hypoxia. At 1 h and 24 h after HI induction, exogenous rh-CSF1 was administered intranasally. To explore the underlying mechanism, CSF1R inhibitor, BLZ945, and phospholipase C-gamma 2 (PLCG2) inhibitor, U73122, were injected intraperitoneally at 1 h before HI induction, respectively. Brain infarct area, brain water content, neurobehavioral tests, western blot, and immunofluorescence staining were performed.

**Results:**

The expressions of endogenous CSF1, CSF1R, PLCG2, protein kinase C epsilon type (PKCε), and cAMP response element-binding protein (CREB) were gradually increased after HIE. Rh-CSF1 significantly improved the neurological deficits at 48 h and 4 weeks after HI, which was accompanied by a reduction in the brain infarct area, brain edema, brain atrophy, and neuroinflammation. Moreover, activation of CSF1R by rh-CSF1 significantly increased the expressions of p-PLCG2, p-PKCε, and p-CREB, but inhibited the activation of neutrophil infiltration, and downregulated the expressions of IL-1β and TNF-α. Inhibition of CSF1R and PLCG2 abolished these neuroprotective effects of rh-CSF1 after HI.

**Conclusions:**

Our findings demonstrated that the activation of CSF1R by rh-CSF1 attenuated neuroinflammation and improved neurological deficits after HI. The anti-inflammatory effects of rh-CSF1 partially acted through activating the CSF1R/PLCG2/PKCε**/**CREB signaling pathway after HI. These results suggest that rh-CSF1 may serve as a potential therapeutic approach to ameliorate injury in HIE patients.

## Introduction

Hypoxic-ischemic encephalopathy (HIE) is a life-threatening cerebrovascular disease which is associated with high morbidity and mortality in infants, resulting in life-long neurodevelopmental impairment, such as cerebral palsy, mental retardation, cognitive deficits, epilepsy, and learning and visual impairments [1–3]. The incidence of HIE in live births is 0.1–0.8% in developed countries, but may vary, with rates as high as 2.6% in undeveloped countries [4]. Currently, therapeutic hypothermia, given shortly after birth, can improve functions and minimize symptoms induced by HIE [5]. However, 50% of infants still suffer from severe neurodevelopmental impairment or even death [6]. The cellular and molecular mechanisms responsible for the pathogenesis of HIE, specifically post-HI neuroinflammation, are complex and remain unclear.

Colony-stimulating factor 1 (CSF1), also known as macrophage-CSF (M-CSF), is a secreted cytokine that is essential to regulate the growth and differentiation of macrophage lineage cells (e.g., macrophages, osteoclasts, and microglia) [7, 8]. In the central nervous system (CNS), CSF1 is primarily expressed on neurons, microglia, astrocytes, and oligodendrocytes [7, 9], which can regulate neuroinflammation, reconstruct damaged white matter, and maintain social behavior [10, 11]. The biological effects of CSF1 are mediated by CSF1 receptor (CSF1R), a unique tyrosine kinase transmembrane receptor [12]. CSF1 binding activates CSF1R through a process of oligomerization and trans-phosphorylation [13]. CSF1R is primarily expressed on microglial cells in the brain [14], with a minimal expression on neurons in the hippocampus and cortex [15]. Neuroinflammation plays a crucial role in the brain injury of neonatal HIE [16]. Microglia are the brain-resident macrophages, and their activation is the initial step in the inflammatory responses of the CNS diseases, such as stroke, Alzheimer’s disease (AD), and experimental autoimmune encephalomyelitis (EAE) [17–19]. CSF1R is essential for microglia viability [15, 20], which not only regulates the development, proliferation, and maintenance of microglia but also plays a major role in neuroinflammation [21, 22]. Moreover, the expression and activity of CSF1R are increased after brain damage [15, 23]. Wlodarczyk et al. reported that stimulation of CSF1R by recombinant mouse CSF1 could induce the expansion of CD11c positive microglia, leading to improvement of EAE symptoms and less demyelination [24]. In the experimental stroke, microglia play neuroprotective roles by phagocytosing neutrophils and inhibiting reactive astrocyte-induced inflammatory processes [25]. Depletion of microglia by CSF1R inhibitor exacerbated ischemic infarction and neurological deficits following ischemic stroke [26]. However, the effects of CSF1R activation have never been investigated in the setting of HIE.

Phospholipase C gamma 2 (PLCG2) belongs to the family of phospholipase C-gamma, which is the downstream molecule of the CSF1R [27]. Previous studies have demonstrated that PLCG2 was required for CSF1-induced differentiation of monocytes, microglia, immune, inflammatory responses [28, 29]. PLCG2 phosphorylation leads to an increase in production of the cellular signaling molecules, diacylglycerol (DAG), and inositol 1,4,5 trisphosphate (IP3), which further promotes a wide range of downstream signals, including the phosphorylation of protein kinase C (PKC) family members, such as PKC epsilon (PKCε) [30, 31]. PKCε, a serine/threonine kinase isoform of the PKC family, exerts wide-scale mitochondrial protection and has an essential neuroprotective function against ischemic injury [32, 33]. It has been reported that PKCε has an anti-inflammatory role in the CNS by regulating the functions of microglia and astrocytes [34, 35]. The activation of PKCε influences a number of signaling mechanisms, such as modulating the phosphorylation of cyclic adenosine monophosphate response element-binding protein (CREB) [36]. CREB has been proved to be a transcription factor that participates in anti-apoptosis, anti-inflammation, and other pathological processes in cerebral ischemia injury, subarachnoid hemorrhage, and HIE [37, 38]. CREB is an important downstream signaling molecule that mediates the biological effects of CSF1 in neurons and macrophages. Recombinant human CSF1 (rh-CSF1) reduced excitotoxin-induced neuronal loss and gliosis via the phosphorylation of CREB, thus attenuating brain injury [15].

In this present study, we explored the role of CSF1 in HIE. We demonstrated, for the first time, that intranasal administration of rh-CSF1 attenuated ischemia-induced neuroinflammation via the CSF1R/PLCG2/PKCε/CREB signaling pathway in a rat model of neonatal HIE (shown in Fig. s1).

## Methods

### Animals

In this study, unsexed Sprague Dawley rat pups were purchased from Envigo Labs (Livermore, CA) with their mothers. Ten-day old rat pups (*n* = 202, weight = 16–22 g) were used. All animals were kept in a 12 h light/dark cycle, in a controlled room environment, with libitum access to breast milk, water, and food. All experimental protocols were approved by the Institutional Animal Care and Use Committee (IACUC) of Loma Linda University, which comply with the National Institutes of Health Guidelines for the Care and Use of laboratory Animals in Neuroscience Research and ARRIVE guidelines.

### HIE model

The animal model of neonatal HIE was performed as previously described [39]. Briefly, rat pups were placed into a temperature-controlled chamber and anesthetized with isoflurane (3% induction, 2.5% maintenance). The temperature was controlled using incubators and a heated blanket during the operative and postoperative period. The rat neck was swabbed with alcohol and draped using standard sterile techniques after anesthesia induction. A small lateral incision (approximately 3–5 mm in length) was made to the right of the midline, across the sagittal plane. Next, the right common carotid artery was isolated and gently separated from its surrounding structures. The right carotid artery was double ligated with 5.0 surgical silk and severed between the ligatures. Gentle pressure was used to control bleeding, and the skin was closed with sutures. All surgeries were completed in 5–9 min. After the surgical procedure, the rats were allowed to recover from anesthesia for 1 h on temperature-controlled heating blankets. Pups were then placed in a 500 ml airtight jar in a 37 °C water bath and were exposed for 2.5 h to a gas mixture of 8% oxygen and 92% nitrogen, which was delivered into the jar via inlet and outlet portals. For the sham animals, the right common carotid artery was subjected to exposure, but without ligation, cutting, or exposure to hypoxic conditions. Thereafter, the animals were returned to their mothers and left in the incubator for 48 h.

### Experimental design

#### Experiment 1

To characterize the time course expressions of endogenous CSF1, CSF1R, PLCG2, PKCε, and CREB after HI, the rats were randomly divided into 7 groups (*n* = 6/group): Sham, 6 h HI, 12 h HI, 24 h HI, 48 h HI, 72 h HI, and 7 d HI. The right (ipsilateral) brain samples were collected for western blot analysis. The rats in the sham group were sacrificed at 24 h after HI.

#### Experiment 2

To evaluate the neuroprotective effects of rh-CSF1 treatment in HIE, the optimal dose of rh-CSF1 treatment for HI injury was tested. Rats were randomly divided into 5 groups (*n* = 6/group): Sham, HI + Vehicle, HI + rh-CSF1 (40 μg/kg), HI + rh-CSF1 (80 μg/kg), and HI + rh-CSF1 (160 μg/kg). Rats were administered intranasally with rh-CSF1 or vehicle (double distilled water, DDH_2_O) at 1 h after HI induction followed by one more injection at 24 h after HI. Infarct volume, brain edema, short-term neurobehavioral tests (negative geotaxis), and body weights were evaluated at 48 h after HI.

#### Experiment 3

To evaluate CSF1 and CSF1R expressions and characterize the expression of exogenous rh-CSF1 in the rat brain after intranasal administration at 48 h after HI. Rats were randomly divided into 3 groups (*n* = 4/group): Sham, HI + Vehicle, and HI + rh-CSF1 (optimal dose). Ionized calcium-binding adapter molecule 1 (Iba-1), a microglia marker, was co-stained with CSF1 or CSF1R to determine colocalization of microglia with CSF1 or CSF1R. Immunofluorescence staining and western blot were conducted to determine the expression of rh-CSF1 in rat brain tissues.

#### Experiment 4

To evaluate the long-term effects of exogenous rh-CSF1 treatment, the rats were randomly divided into 3 groups (*n* = 8/group): Sham, HI + Vehicle, and HI + rh-CSF1 (optimal dose). Neurobehavioral tests including Foot-fault, Rotarod, and Morris water maze were performed at 4 weeks after HI, after which the rats were sacrificed. The brains were removed and cut into ipsilateral and contralateral hemispheres, which were then weighed separately, and subsequently prepared for Nissl staining.

#### Experiment 5

To assess whether CSF1R was involved in the underlying mechanisms of rh-CSF1-mediated neuroprotective effects, the CSF1R inhibitor, BLZ945, was used to inhibit CSF1R. Rats were randomly divided into 5 groups (*n* = 6/group): Sham, HI + vehicle (vehicle of rh-CSF1, DDH_2_O), HI + rh-CSF1, HI + rh-CSF1 + dimethyl sulfoxide (DMSO) (Vehicle of BLZ945), and HI + rh-CSF1 + BLZ945. Rh-CSF1 (optimal dose) or DDH_2_O were administered intranasally at 1 h and 24 h after HI induction. BLZ945 or DMSO was injected intraperitoneally at 1 h before HI induction. Infarct volume, brain edema, negative geotaxis, body weight, western blot, and immunofluorescence staining were examined at 48 h after HI.

#### Experiment 6

To assess whether PLCG2 was involved in the underlying mechanisms of rh-CSF1-mediated neuroprotective effects, U73122 was used to inhibit the activity of PLCG2. Rats were randomly divided into 5 groups (*n* = 6/group, shared with Experiment 5 except for the group of HI+rh-CSF1+U73122): Sham, HI + vehicle (vehicle of rh-CSF1, DDH_2_O), HI + rh-CSF1, HI + rh-CSF1 + DMSO (Vehicle of U73122), and HI + rh-CSF1 + U73122. Rh-CSF1 (optimal dose) or DDH_2_O was administered intranasally at 1 h and 24 h post-HI induction. U73122 (30 mg/kg) or DMSO was injected intraperitoneally at 1 h before HI. Infarct volume, brain edema, negative geotaxis, body weight, western blot, and immunofluorescence staining were examined at 48 h post-HI.

The rectal temperature, heart rate, and respiration rate of each group were measured in Experiment 2, 5, and 6.

### Drug administration

Rh-CSF1 (40, 80, 160 μg/kg, Abcam, USA) or vehicle of rh-CSF1 (DDH_2_O) was administered intranasally at 1 h and 24 h after HI. A total of 5 μl of rh-CSF1 or DDH_2_O was given every 2 min in alternating nares. BLZ945 (60 mg/kg, Cayman chemical, USA), U73122 (30 mg/kg, Cayman chemical, USA), or DMSO was injected intraperitoneally at 1 h before HI.

### Measurement of infracted area

At 48 h after HI, the rat pups were anesthetized and euthanized, followed by immediate transcardiac perfusion using 20 mL of chilled phosphate-buffered saline (PBS, 0.01 M, pH 7.4). The brains were removed, sectioned into slices (2 mm), and stained with 2% solution 2,3,5-triphenyltetrazolium chloride monohydrate (TTC) (Sigma Aldrich Inc., USA) for 5 min and then washed in PBS [40]. The brain slices were digitally photographed to outline and the total area of contralateral hemispheres and non-infarcted area of ipsilateral hemispheres for each slice was measured using Image J software (NIH, USA). The infarcted areas were calculated using the following formula: [(total area of contralateral hemisphere) − (area of un-infarcted area of ipsilateral hemisphere)] / (total area of contralateral hemisphere × 2). The average value was taken to represent the percentage of infarcted area for that animal.

### Brain water content

Brain water content was performed as previously described [41]. The rat pups were anesthetized and euthanized at 48 h after HI, and the brain hemispheres were quickly removed and separated into ipsilateral and contralateral hemispheres. The right (ipsilateral) cerebral hemispheres were weighed immediately to obtain the wet weight and then dried at 100°C for 48 h. The dried brain was re-weighed. The percentage of brain water content was calculated as (wet weight − dry weight) /wet weight × 100%.

### Neurological evaluation

Negative geotaxis test was performed in a blinded setup at 48 h after HI for evaluating short-term neurological function. Foot-fault, Rotarod, and Morris water maze tests were performed in a blinded setup at 4 weeks after HI for evaluating long-term neurological function.

#### Negative geotaxis test

Negative geotaxis test was performed by placing rat pups on a sloping board (45°) with their head facing downward. Next, the time was recorded to determine the length of time it took for the pups to rotate their body so that they are facing upward. The maximum testing time was 60 s, and any recordings exceeding 60 s were documented as 60 s.

#### Rotarod test

The Rotarod test was performed by placing the rat pups on a rotating horizontal rod (Columbus Instruments Rotamex, USA), then the times were recorded when the rats fell. The rotation speed started from 5 or 10 rpm separately and accelerated 2 rpm every 5 s. The maximum testing time was 60 s, and any times exceeding 60 s were recorded as 60 s [42].

#### Foot-fault test

The Foot-fault test was performed by placing the rat pups on a horizontal grid floor (square size 20–40 cm with a mesh size of 4 cm^2^) elevated 1 m above ground for 1 min. A Foot-fault was defined as when the animal inaccurately placed a forelimb or hindlimb, causing the paw to fall down between the grid bars. The number of foot-faults for each rat was recorded using video equipment and analyzed by an investigator blind to the experimental groups.

#### Morris water maze test

The Morris water maze test was performed on days 23–27 after HI to evaluate spatial learning capacity and memory, as previously shown [43]. A hidden platform was submerged in a pool of water using visual cues around the room. The rats underwent a 6-day test, consisting of 5 trials in both cued and hidden tests over 5 days, and a probe trial being conducted on the final day. None of the trials lasted more than 60 s, a 10-min interval between consecutive trials was allotted for rest. On day 1, the rats were trained using a visible platform in the cued test (block 1). If the rats had not found the platform in 60 s, they were manually guided to the platform. On days 2–5, the rats were required to find a platform submerged 1 cm below the water in the memory test (blocks 2-5), if the rats had not discovered the platform in 60 s, they were manually guided to the platform. On day 6, the platform was removed in the probe trial, and the time that each rat took in the platform quadrant was measured (block 6). A video recording system-2000 (San Diego Instruments Inc, USA) traced all of the animals’ activities and the swim paths to quantify the total distance of swimming, latency to reach the platform and swimming speed.

### Western blotting analysis

Western blot was processed as described previously [44]. After TTC staining and images were recorded at 48 h after HI, the brain slices were divided into ipsilateral and contralateral cerebrums and stored immediately in a −80 °C freezer for future use. The right/ipsilateral hemisphere tissue was homogenized in RIPA lysis buffer (Santa Cruz Biotechnology, USA) with protease inhibitor cocktail for 15 min and then centrifuged at 14,000*g* at 4 °C for 30 min. The supernatant was collected, and protein quantification was performed using a detergent compatible assay (Bio-Rad, DC™ Protein Assay). Equal amounts of protein were loaded onto a 10%~12% sodium dodecyl sulfate-polyacrylamide gel and electrophoresed. Next, they were transferred onto nitrocellulose membranes, which were blocked with 5% non-fat blocking grade milk (Bio-Rad, Hercules, USA) and incubated at 4 °C with the following primary antibodies: anti-CSF1 (reacts with human and rats, 1:1000, Abcam, USA), anti-CSF1 (reacts specifically with human, 1:500, Thermo Fisher Scientific, USA), anti-CSF1R (1:500, LSBio, USA), anti-p-CSF1R (1:1000, Thermo Fisher Scientific, USA), anti-PLCG2 (1:500, Novus biologicals, USA), anti-p-PLCG2 (1:1000, Abcam, USA), anti-PKCε (1:1000, Abcam, USA), anti-p-PKCε (1:1000, Abcam, USA), anti-CREB (1:1000, Abcam, USA), anti-p-CREB (1:1000, Abcam, USA), anti-interleukin (IL) -1β (1:1000, Abcam, USA), TNF-α (1:1000, Abcam, USA) and Goat anti-β-actin (1:3000, Santa Cruz Biotechnology, USA). Then, the appropriate secondary antibodies (1:3000, Santa Cruz Biotechnology, USA) were incubated at room temperature for 2 h. The membranes were probed with an ECL Plus chemiluminescence reagent kit (Amersham Biosciences, USA). The specific bands were visualized by an ECL Plus chemiluminescence reagent kit (Amersham Biosciences, USA). The relative density of protein was analyzed using Image J software (NIH, USA).

### Histological analysis

Rat pups were transcardially perfused under deep anesthesia with cold PBS and 10% formalin at 2 days or 28 days after HI. The brains were removed and fixed in 10% formalin for 24 h, and then stored in a 30% sucrose solution at 4 °C until they sank. After being embedded into OCT compound (Scigen Scientific, USA) and frozen, the coronal sections were cut sequentially at 8–10 μm thickness for immunofluorescence (2 days after HI) and 15–20 μm for Nissl staining (28 days after HI) at −20 °C with a cryostat (LM3050S; Leica Microsystems, Germany).

### Immunofluorescence staining

Immunofluorescence staining was performed as described previously [45]. The sections were permeabilized with 0.3% Triton X-100 for 10 min and then blocked with 5% donkey serum for 2 h at room temperature. Subsequently, sections were incubated at 4 °C overnight with primary antibodies: Iba-1 (1:200, Abcam, USA), anti-CSF1 (1:100, Abcam, USA), anti-CSF1R (1:100, LSBio, USA), IL-1β (1:200, Abcam, USA), myeloperoxidase (MPO) (1:200, Abcam, USA). The following day, the sections were incubated with the appropriate fluorescence-conjugated secondary antibodies (1:150) for 1 h at room temperature and mounted using Vectashield Antifade Mounting Medium with DAPI (Vector Laboratories Inc., USA). The stained sections were then captured under a fluorescence microscope (Leica DMi8, Leica Microsystems, Germany), and the staining positive cells were counted and averaged from the 5 fields of view within peri-lesion area under the microscope.

### Nissl staining

Nissl staining was performed to evaluate brain tissue loss. The sections were successively dehydrated in 95% and 70% ethanol for 2 min and then stained with 0.5% cresyl violet (Sigma-Aldrich, USA) for 2 min, followed by dehydration with 100% ethanol and xylene for 2 min twice, respectively, before the sections were mounted with DPX (Sigma-Aldrich, USA). The sections were imaged using a microscope (Olympus-BX51) equipped with MagnaFire SP 2.1B software (Olympus). A total of 3 slices were used from each brain to determine the average percent of tissue loss, which was measured using Image J software (NIH, USA). The percentage of brain tissue loss was calculated using the same equation for the infarct area. Brain weight loss was calculated using the following formula: weight of contralateral hemisphere-weight of the ipsilateral hemisphere.

### Statistical analysis

The data were presented as mean ± SD and plotted using Graph Pad Prism 8 (Graph Pad Software, USA). Statistical analysis was performed with SPSS v. 21.0 software (IBM, USA). Single-factor analysis of variance (one-way ANOVA)) was used followed by multiple comparisons among groups using the Tukey post hoc test. Student’s *t* test was used to compare the differences between the two groups. Differences with *P* < 0.05 were considered statistically significant.

## Results

### Expression levels of endogenous p-CSF1R, CSF1R, CSF1, p-PLCG2, p-PKCε, and p-CREB were upregulated in a time-dependent manner after HI

**Fig. 1:**
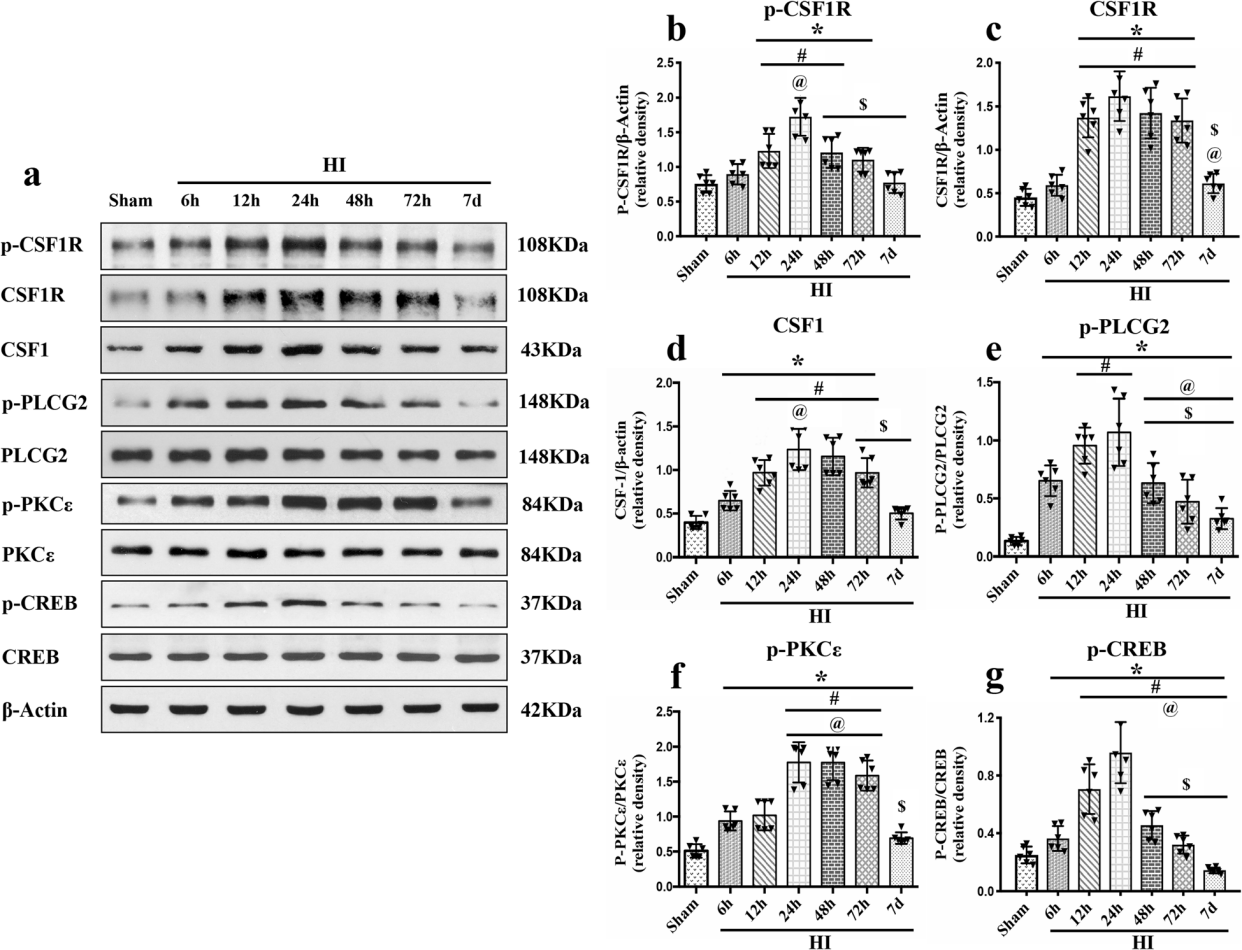
Temporal expression of endogenous p-CSF1R, CSF1R, CSF1, p-PLCG2, p-PKCε, and p-CREB in the ipsilateral hemisphere brain after HI. **a** Representative western blot bands of the time course. **b-g** Western blot data analysis showed that endogenous expression levels of p-CSF1R, CSF1R, CSF1, p-PLCG2, p-PKCε, and p-CREB were upregulated over 7 d after HI, starting at 6 h and peaking at 24 h after HI. *P < 0.05 vs sham; ^**#**^*P* < 0.05 vs 6 h HI; ^**@**^*P* < 0.05 vs 12 h HI; ^**$**^*P* < 0.05 vs 24 h HI. Data are represented as mean ± SD, *n* = 6 in each group

As shown in Fig. 1, the endogenous expression levels of p-CSF1R, CSF1R, CSF1, p-PLCG2, p-PKCε, and p-CREB were upregulated after HI, and peaked at 24 h when compared with the sham group (*P* < 0.05).

### Intranasal administration of exogenous rh-CSF1 reduced infarcted area, brain edema, improved short-term neurological deficits and reduced body weight loss at 48 h after HI

**Fig. 2:**
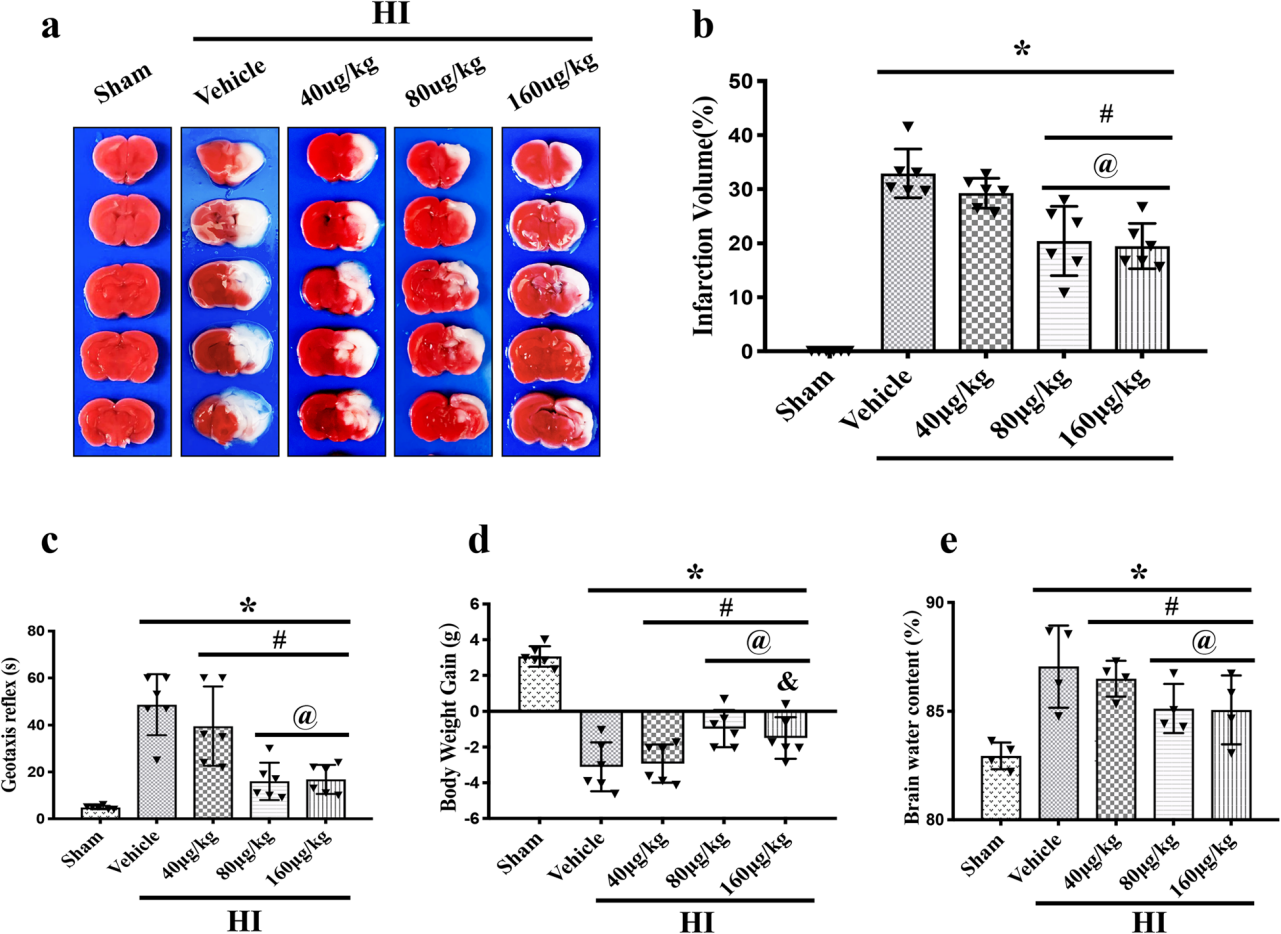
Effect of intranasal administration of rh-CSF1 on brain infarcted area, brain edema, short-term neurological function and body weight at 48 h after HI. **a–b** TTC staining showed that medium (80 μg/kg) and high (160 μg/kg) dose of rh-CSF1 significantly reduced the infarcted area when compared with the low (40 μg/kg) dose and vehicle. **c** Geotaxis reflex test showed that all HI animals had a significantly longer reflex time than the sham group. All three doses of rh-CSF1 treatments significantly improved geotaxis reflex performance compared with the vehicle-treated HI animals. In addition, the medium (80 μg/kg) and high (160 μg/kg) doses of rh-CSF1 resulted in a significantly shorter reflex time than the low (40 μg/kg) dose. **d** All HI animals showed significant weight loss compared with the sham group. All three doses of rh-CSF1 treatment significantly reduced the weight loss with the best effects in medium-dose group (80 μg/kg). **e** All HI animals showed significant brain water content increase compared with the sham group. The medium (80 μg/kg) and high (160 μg/kg) doses of rh-CSF1 treatment significantly reduced brain edema compared with the vehicle and low (40 μg/kg) dose-treated HI animals. *P < 0.05 vs sham; ^**#**^*P* < 0.05 vs vehicle; ^**@**^*P* < 0.05 vs rh-CSF1 (40 μg/kg); ^**&**^*P* < 0.05 vs rh-CSF1 (80 μg/kg); Data are represented as mean ± SD, *n* = 6 in each group

TTC staining (Fig. 2a and b) revealed that no significant differences were found regarding the infarcted area in the low dose group (40 μg/kg) when compared with the vehicle group (*P* > 0.05). Medium (80 μg/kg) and high (160 μg/kg) doses of rh-CSF1 treatment significantly reduced the percentage of the infarcted area when compared with the low dose and vehicle groups (*P* < 0.05).

Negative geotaxis tests (Fig. 2c) showed that pups spent more time rotating to the upward head position when compared to the sham group at 48 h after HI (*P* < 0.05). Rh-CSF1 significantly improved neurological function compared with the vehicle-treated animals. Medium and high doses of rh-CSF1 decreased the reflex time significantly when compared with the low dose and vehicle groups (*P* < 0.05).

Body weight measurement results (Fig. 2d) showed that three doses of rh-CSF1 dose treatments (40, 80, and 160 μg/kg) significantly decreased body weight loss when compared with the vehicle group at 48 h after HI (*P* < 0.05), of which the medium dose group (80 μg/kg) showed the best performance when compared to the other two doses groups (*P* < 0.05). Thus, 80 μg/kg of rh-CSF1 was selected as the optimal dose and used for the remaining experiments.

Brain water content (Fig. 2e) showed that no significant difference was found regarding the brain edema in the low dose group (40 μg/kg) when compared with the vehicle group (*P* > 0.05). Medium (80 μg/kg) and high (160 μg/kg) doses of rh-CSF1 treatment significantly reduced brain edema when compared with the low dose and vehicle groups (*P* < 0.05).

### Intranasal administration of exogenous rh-CSF1 resulted in the expression of human CSF1 in the rat brain at 48 h after HI

**Fig. 3:**
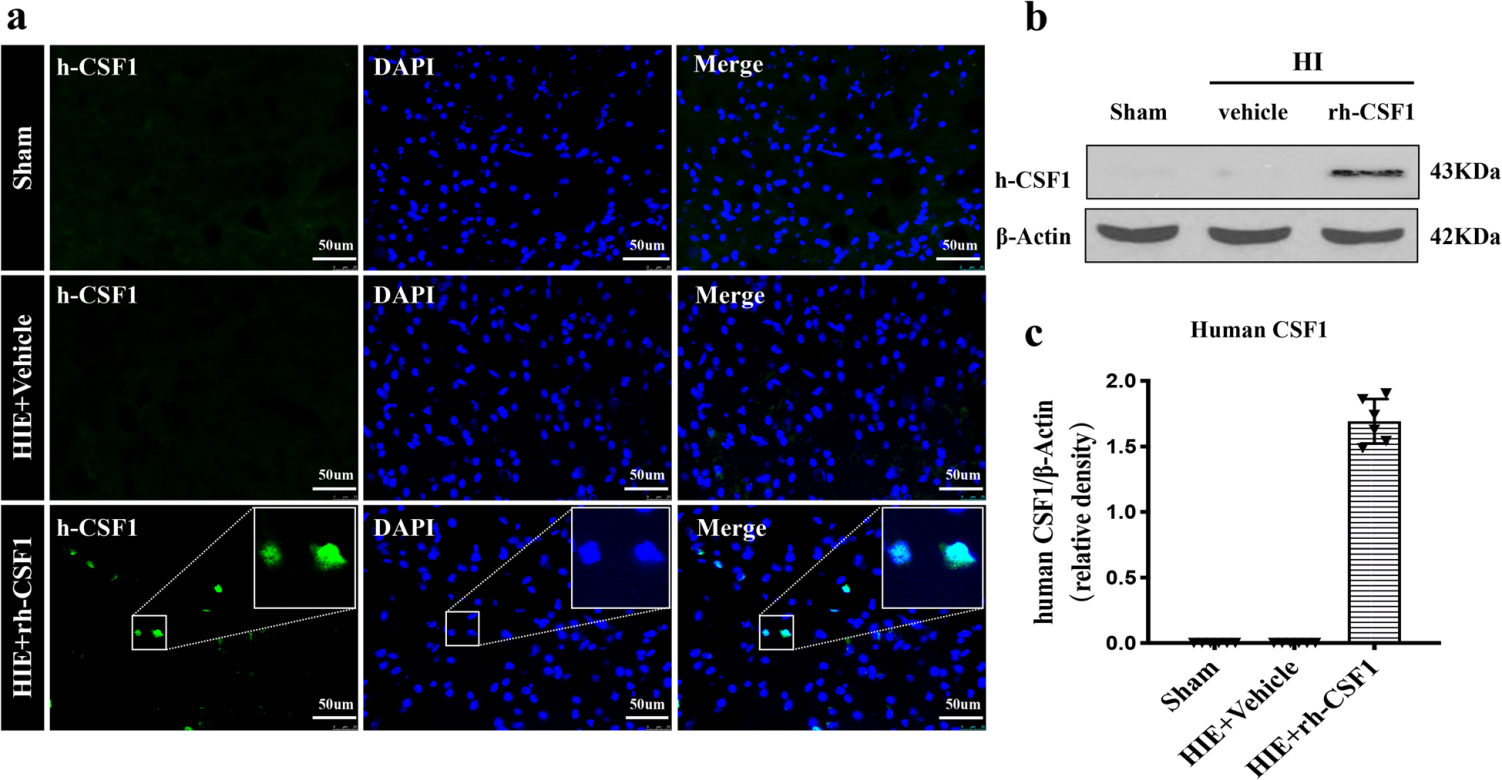
Expression of exogenous rh-CSF1 in rat brain after intranasal administration at 48 h after HI. **a** Representative microphotographs of immunofluorescence staining depicted the human CSF1 expression in the rat brain after the intranasal administration of exogenous rh-CSF1. **b** Representative western blot bands showed the expression of human CSF1 in the rat brain after intranasal administration of exogenous rh-CSF1. **c** Quantitative analysis of western blot bands showed that there was a significant amount of human CSF1 in the rat brain after intranasal administration of exogenous rh-CSF1. Data are represented as mean ± SD, *n* = 6 in each group

Immunofluorescence staining depicted the fluorescence staining of human CSF1 on the cell after intranasal administration of exogenous rh-CSF1 at 48 h after HI (Fig. 3a), and western blot data showed the bands of human CSF1 in the rat brain (Fig. 3b, c), but not in the sham group or vehicle groups. These results illustrated that exogenous rh-CSF1 could enter the rat brain tissue via the intranasal administration route.

### Double immunofluorescence staining showed the colocalization of CSF1 and CSF1R with microglia at 48 h after HI

**Fig. 4:**
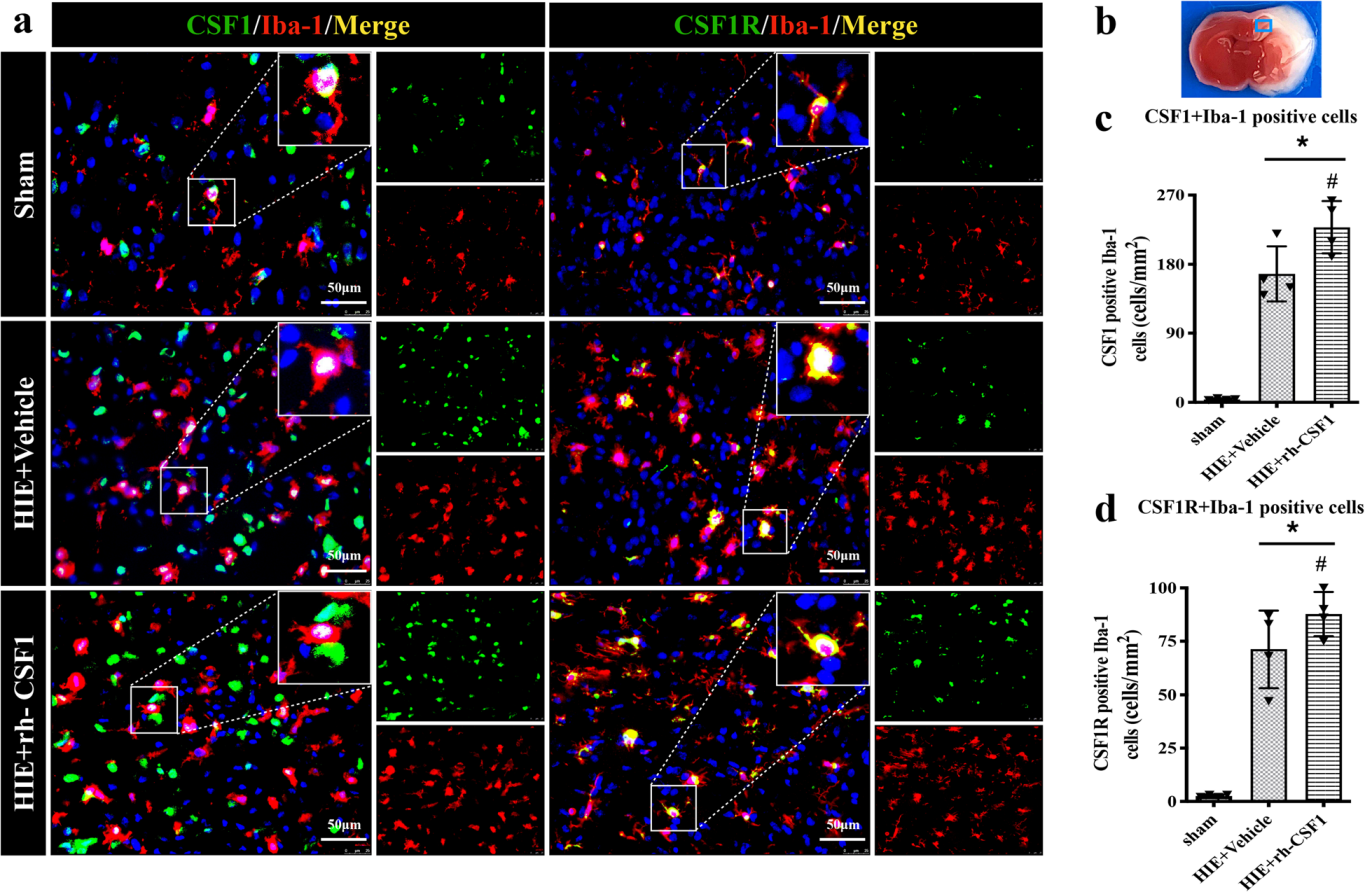
Double immunofluorescence staining of CSF1 and CSF1R with Iba-1 in rat brain at 48 h after HI. **a** Representative microphotographs of CSF1 and CSF1R and Iba-1 staining in the ipsilateral cortex of rat brain. CSF1 and CSF1R were colocalized with Iba-1 positive microglia in the sham, vehicle, and rh-CSF1 treatment groups, respectively. **b** Brain slice of rats at 48 h after HI. The upper right panel of brain slice indicates the location for staining analysis (small blue box). **c, d** Quantitative analysis of CSF1-positive Iba-1 and CSF1R-positive Iba-1 cells in the ipsilateral cortex at 48 h after HI. Compared with the sham group, immunofluorescence staining showed an increase in expression of CSF1 and CSF1R on microglia in the vehicle group and rh-CSF1-treated animals. Intranasal administration of rh-CSF1 further increased the expression of CSF1 and CSF1R on microglia than that of the vehicle group. Green indicated CSF1 and CSF1R positive staining, red indicated Iba-1 positive microglia staining, and blue indicated DAPI positive nuclear staining. Merge showed the colocalization of CSF1 and CSF1R with microglia. *P < 0.05 vs sham; ^**#**^*P* < 0.05 vs vehicle; Scale bar =50 μm; *n* = 4 in each group

Double immunofluorescence staining showed that CSF1 and CSF1R colocalized with the Iba-1 positive microglial at 48 h after HI. The expression of CSF1 and CSF1R on microglia was increased after HI including the vehicle group and rh-CSF1-treated animals when compared with the sham group. Intranasal administration of rh-CSF1 promoted proliferation and activation of microglia and further increased the expression of CSF1 and CSF1R on microglia when compared to the vehicle group (Fig. 4).

### Rh-CSF1 reduced neuroinflammation at 48 h after HI

**Fig. 5:**
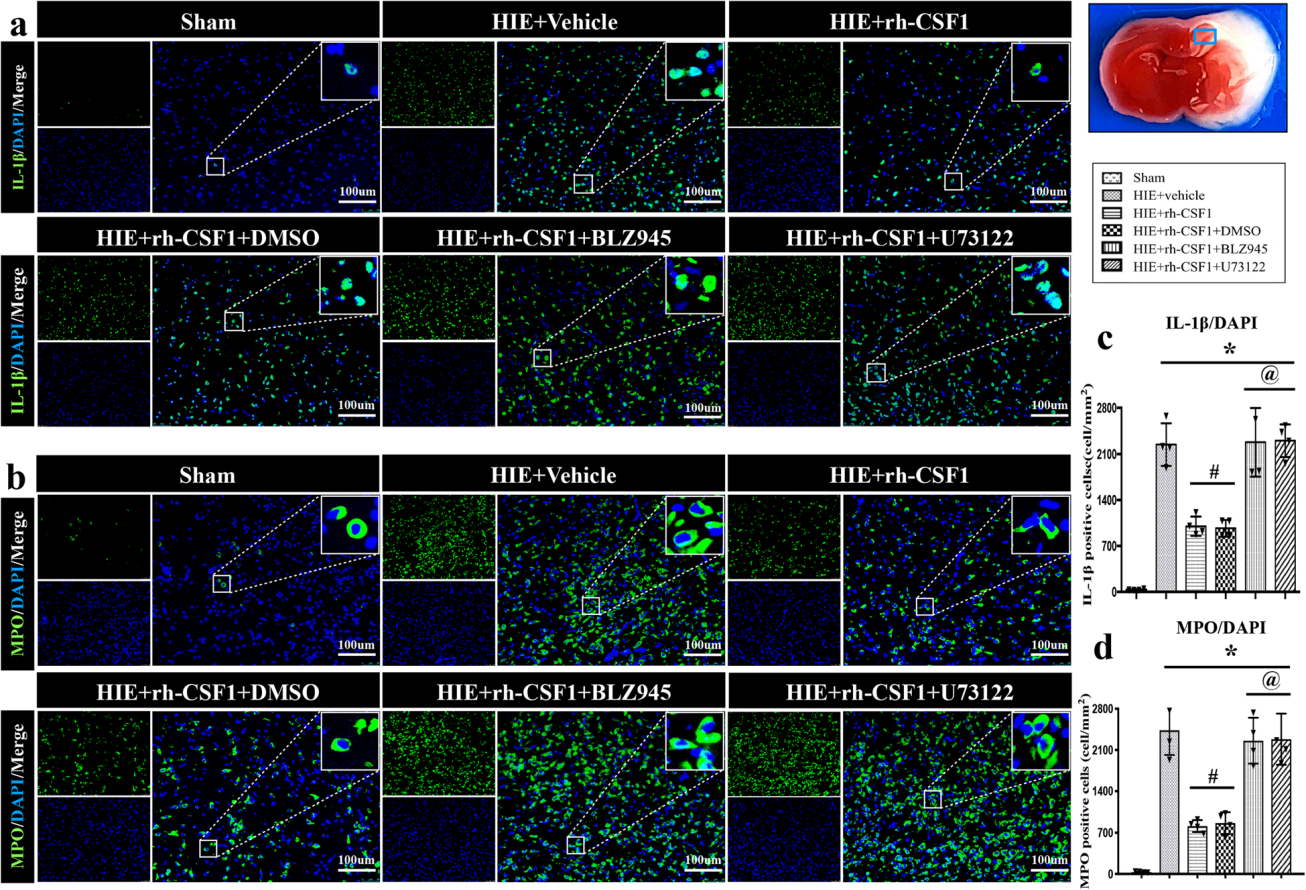
Effects of BLZ945 and U73122 co-administration with rh-CSF1 on immunofluorescence staining of MPO and IL-1β at 48 h after HI. Representative microphotographs **(a-b)** and quantification of **(c-d)** IL-1β and MPO positive cells showed the number of IL-1β/MPO-positive cells was significantly increased in all HI animals compared to shams. Rh-CSF1 treatment or Rh-CSF1 with DMSO significantly reduced the neuroinflammation, but such effects were reversed by either BLZ945 or U73122. *P < 0.05 vs. sham; ^**#**^*P* < 0.05 vs. HI+ vehicle; ^**@**^*P* < 0.05 vs. HI+ rh-CSF1 + DMSO; Scale bar =100 μm; Data are represented as mean ± SD, *n* = 4 in each group

Immunofluorescence staining of MPO and IL-1β were performed to evaluate the neuroinflammation. As shown in Fig. 5, the number of IL-1β/MPO-positive cells in the vehicle group was higher compared to the sham group at 48 h after HI, while intranasal administration of rh-CSF1 significantly reduced IL-1β/MPO-positive cells when compared to the vehicle group.

### Rh-CSF1 improved long-term neurological deficits and reduced brain atrophy at 4 weeks after HI

**Fig. 6:**
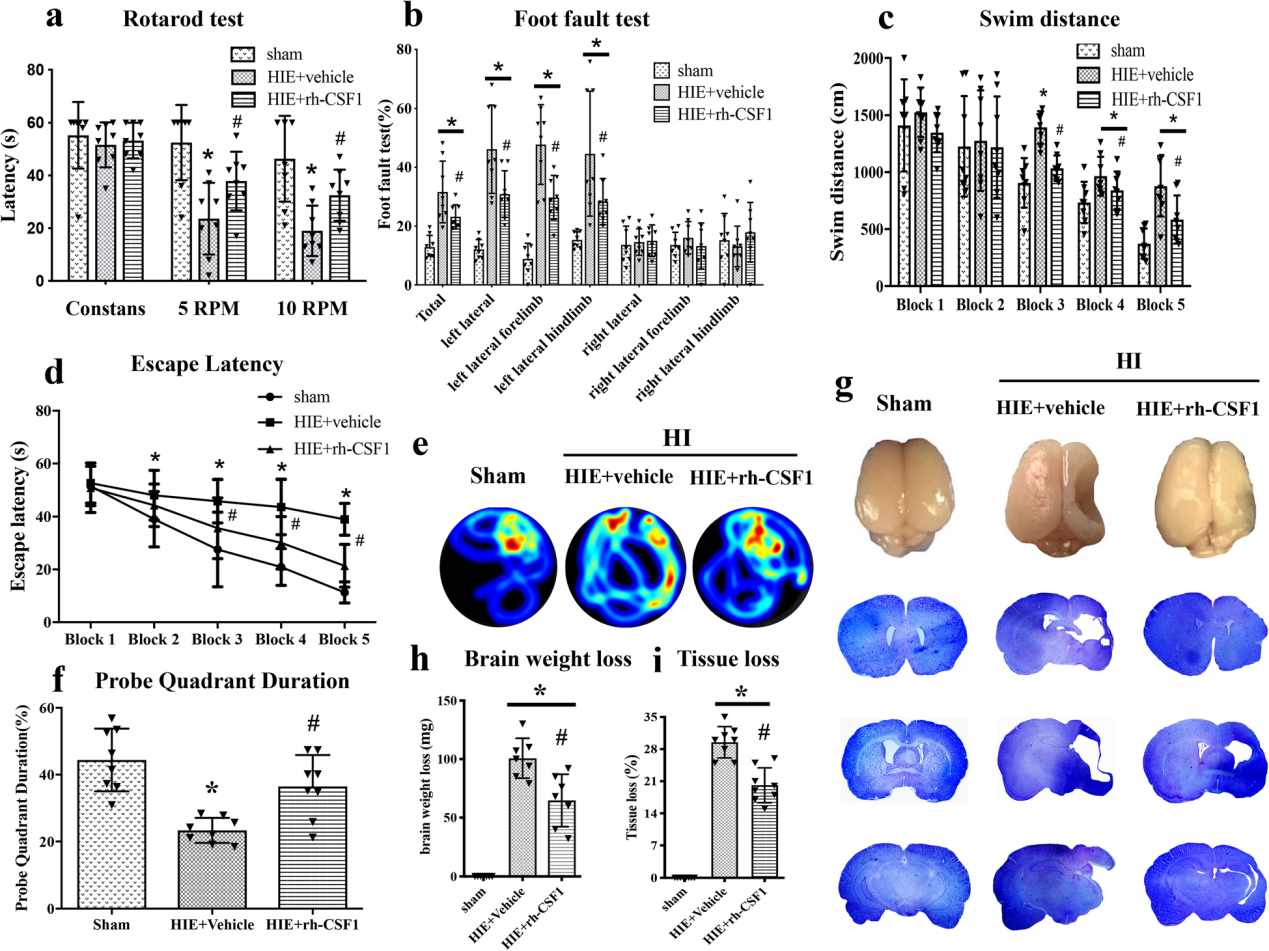
Effects of rh-CSF1 on brain atrophy and neurological functions at 4 weeks post-HI. Rh-CSF1 treatment group showed significant improvement in the rotarod test **(a)**, foot fault test **(b)**, and spatial memory loss in terms of swimming less distance to find the platform **(c)** when compared with the vehicle. Rh-CSF1 treatment group also showed a decrease in escape latency **(d),** and more time spent in the target quadrant during the probe trial **(e-f)** when compared with the vehicle. **f** Representative picture of swim track in Probe Trial. Representative microphotograph of Nissl staining showed brain tissue loss in the ipsilateral hemisphere **(g)**. Rh-CSF1 treatment significantly reduced brain weight loss and the percent of tissue loss **(h-i)** when compared with the vehicle. *P < 0.05 vs sham; ^**#**^*P* < 0.05 vs vehicle. Data are represented as mean ± SD, *n* = 8 in each group

In the rotarod test, animals in the vehicle group had significantly shorter falling latency at both 5 rpm and 10 rpm accelerating velocity tests when compared with the sham group at 4 weeks after HI. However, rh-CSF1 treatment markedly improved the rotarod performance of HIE rats at both 5 rpm and 10 rpm velocities when compared with the vehicle group (*P* < 0.05, Fig. 6a).

In the foot-fault test, animals in the vehicle group displayed more foot-faults on the contralateral side (left) when compared with the sham group at 4 weeks after HI. However, rh-CSF1 treatment markedly improved the foot-faults performance when compared with the vehicle group (*P* < 0.05, Fig. 6b).

In the water maze test, animals in the vehicle group swam a significantly greater distance in 1 min (Fig. 6c), had longer escape latency (Fig. 6d), and required more time to find the platform in the target quadrant during the probe trial (Fig. 6e, f) when compared with the sham group (*P* < 0.05). However, rh-CSF1 treatment improved memory and learning abilities with the decreased total swimming distance (Fig. 6c) and shorter time to find the platform (Fig. 6d), as well as more time spent in the platform quadrant (Fig. 6e, f) when compared with the vehicle group (*P* < 0.05).

Nissl staining was conducted to assess the brain atrophy at 4 weeks after HI. HI caused severe brain damage, characterized by significant brain tissue loss and brain weight loss of the ipsilateral hemisphere, while intranasal administration of rh-CSF1 significantly attenuated brain tissue loss and brain weight loss when compared with the vehicle group (*P* < 0.05, Fig. 6 g, h, i).

### Inhibition of CSF1R and PLCG2 reversed the neuroprotective effects of rh-CSF1 at 48 h after HI

**Fig. 7:**
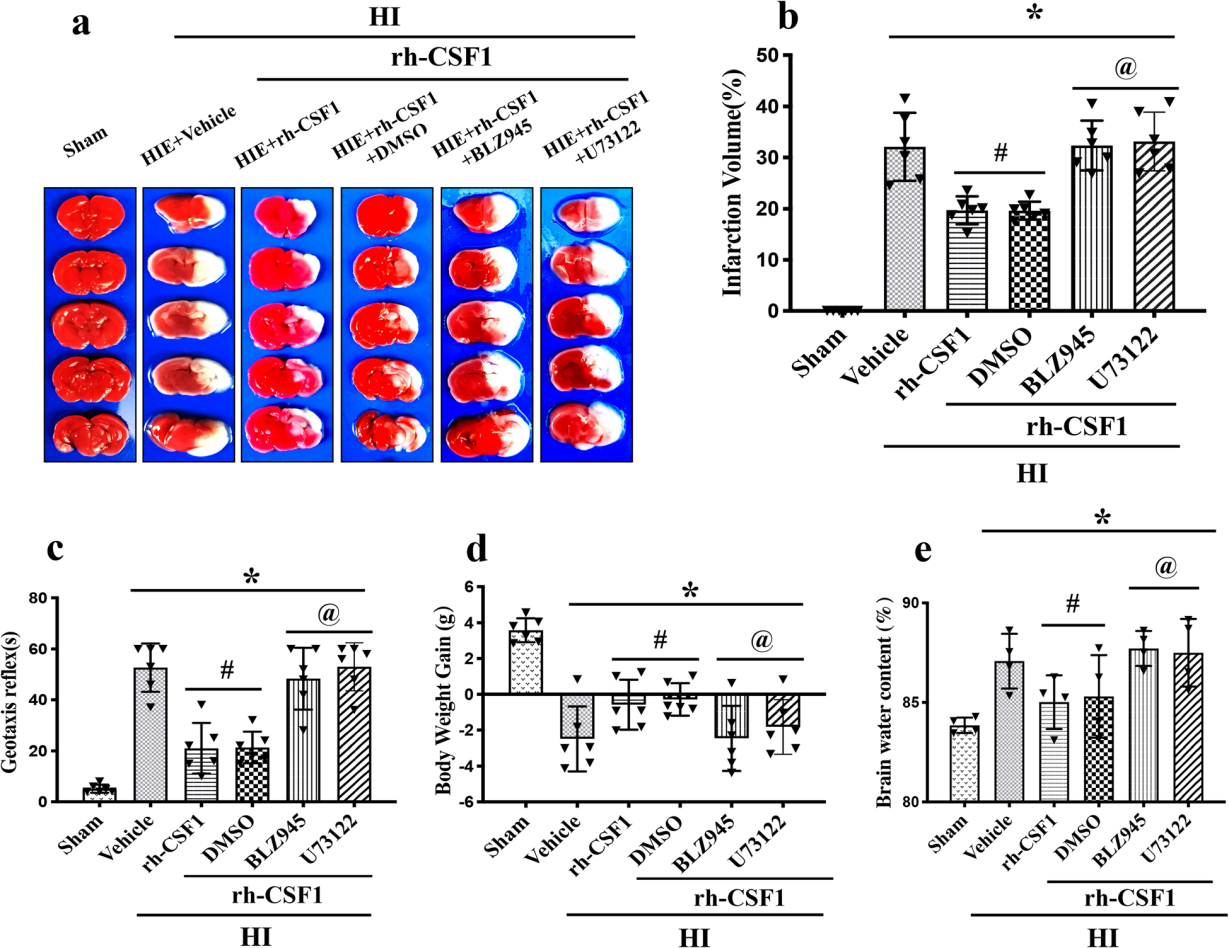
Effects of CSF1R inhibitor BLZ945 and PLCG2 inhibitor U73122 co-administration with rh-CSF1 on infarction volume, brain edema, body weight, geotaxis reflex at 48 h after HI. **a-b** All HI animals have a significantly greater percentage of infarction compare to the sham group. Rh-CSF1 treatment or Rh-CSF1 with DMSO significantly reduced the infarction area, but such effects were reversed by either BLZ945 or U73122. **c** Geotaxis reflex performance was significantly worse in all HI animals compared to the sham group. Rh-CSF1 treatment or Rh-CSF1 with DMSO treatment groups improved this neurological deficit. However, either BLZ945 or U73122 significantly abolished the neurological benefit of rh-CSF1 treatment. **d** All of HI animals had significant body weight loss than shams. Rh-CSF1 treatment or Rh-CSF1 with DMSO significantly attenuated HI-induced body weight loss, which were reversed by BLZ945 or U73122. **e** All HI animals showed significant brain water content increase compared with the sham group. Rh-CSF1 treatment or Rh-CSF1 with DMSO significantly reduced HI-induced brain edema, which were reversed by BLZ945 or U73122. *P < 0.05 vs. sham; ^#^*P* < 0.05 vs. HI+ vehicle; ^@^*P* < 0.05 vs. HI+ rh-CSF1+ DMSO. Data are represented as mean ± SD, *n* = 6 in each group

To evaluate whether CSF1 exerts its neuroprotective effects via the CSF1R/PLCG2/PKCε/CREB signaling pathway, we used BLZ945 (the specific CSF1R inhibitor) and U73122 (the specific PLCG2 inhibitor) with intranasal rh-CSF1 treatment. Both inhibitors reversed the neuroprotective effects of rh-CSF1, resulted in an increase in the percentage of the infarcted area and brain edema when compared with the corresponding control groups (*P* < 0.05, Fig. 7a, b, e). The Negative geotaxis test showed that rats treated with either CSF1R or PLCG2 inhibitors with rh-CSF1 had significantly impaired neurological function when compared with the corresponding rh-CSF1 treatment control groups (*P* < 0.05, Fig. 7c). Consistently, there was a significant change in body weight in the animals treated by intraperitoneally injected with CSF1R or PLCG2 inhibitor with intranasal rh-CSF1 when compared with corresponding treatment control groups (*P* < 0.05, Fig. 7d). In addition, the intensity levels of MPO and IL-1β in both inhibitor groups were much higher compared to the corresponding treatment control groups at 48 h after HI (*P* < 0.05, Fig. 5).

### Inhibition of CSF1R and PLCG2 reversed the anti-neuroinflammation effect of rh-CSF1 through the CSF1R/PLCG2/PKCε/CREB signaling pathway at 48 h after HI

**Fig. 8:**
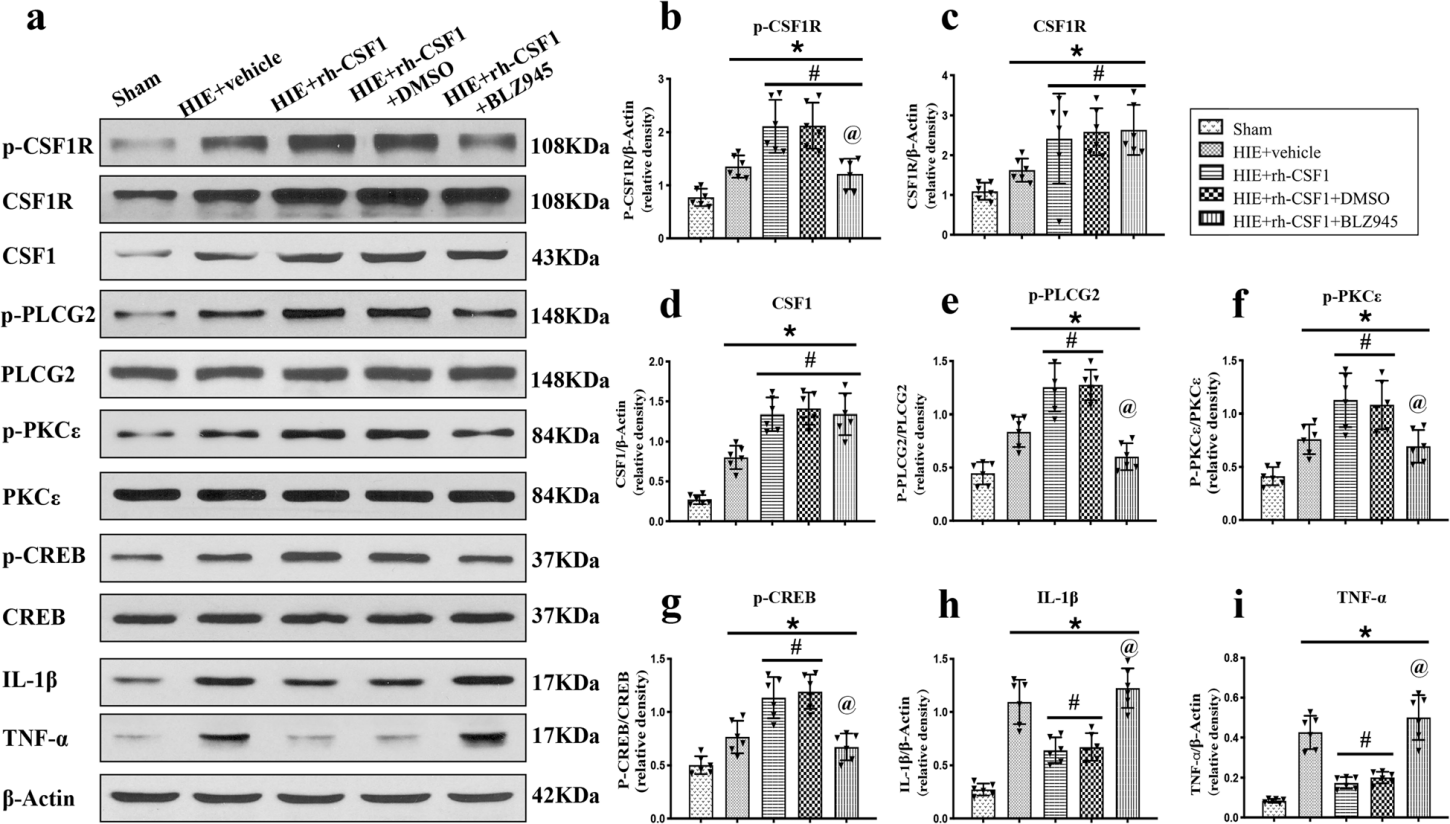
CSF1R inhibitor BLZ945 abolished the anti-inflammatory effects of rh-CSF1 via CSF1R/PLCG2/PKCε/CREB signaling pathway at 48 h after HI. **a** Representative western blot bands. **b–i** Densitometric quantification of p-CSF1R, CSF1R, CSF1, p-PLCG2/PLCG2, p-PKCε/PKCε, p-CREB/CREB, IL-1β and TNF-α in the ipsilateral hemisphere showed that rh-CSF1 treatment upregulated the levels of p-CSF1R, p-PLCG2, p-PKCε, and p-CREB, leading to less pro-inflammatory cytokines of IL-1β and TNF-α. BLZ945 significantly abolished such effects when administered with rh-CSF1. *P < 0.05 vs. sham; ^**#**^*P* < 0.05 vs. HI+ Vehicle; ^**@**^*P* < 0.05 vs. HI+ rh-CSF1 + DMSO; Data are represented as mean ± SD, *n* = 6 in each group

Western blot data showed that all target proteins and critical inflammatory cytokines, including CSF1, total-CSF1R, p-CSF1R, p-PLCG2, p-PKCε, p-CREB, IL-1β, and TNF-α were upregulated at 48 h after HI when compared with the sham group. However, after rh-CSF1 treatment, the expressions of CSF1, total-CSF1R, p-CSF1R, p-PLCG2, p-PKCε, and p-CREB further increased (*P* < 0.05, Fig. 8a-g), but the expression of pro-inflammatory IL-1β and TNF-α were decreased (*P* < 0.05, Fig. 8 h-i) when compared with the vehicle group. CSF1R inhibitor significantly decreased p-CSF1R expression, thereby abolishing the effects of rh-CSF1, evidenced by the attenuated levels of p-PLCG2, p-PKCε, and p-CREB, and increased expression levels of IL-1β and TNF-α compared with the DMSO group at 48 h after HI (*P* < 0.05, Fig. 8a-i).

**Fig. 9:**
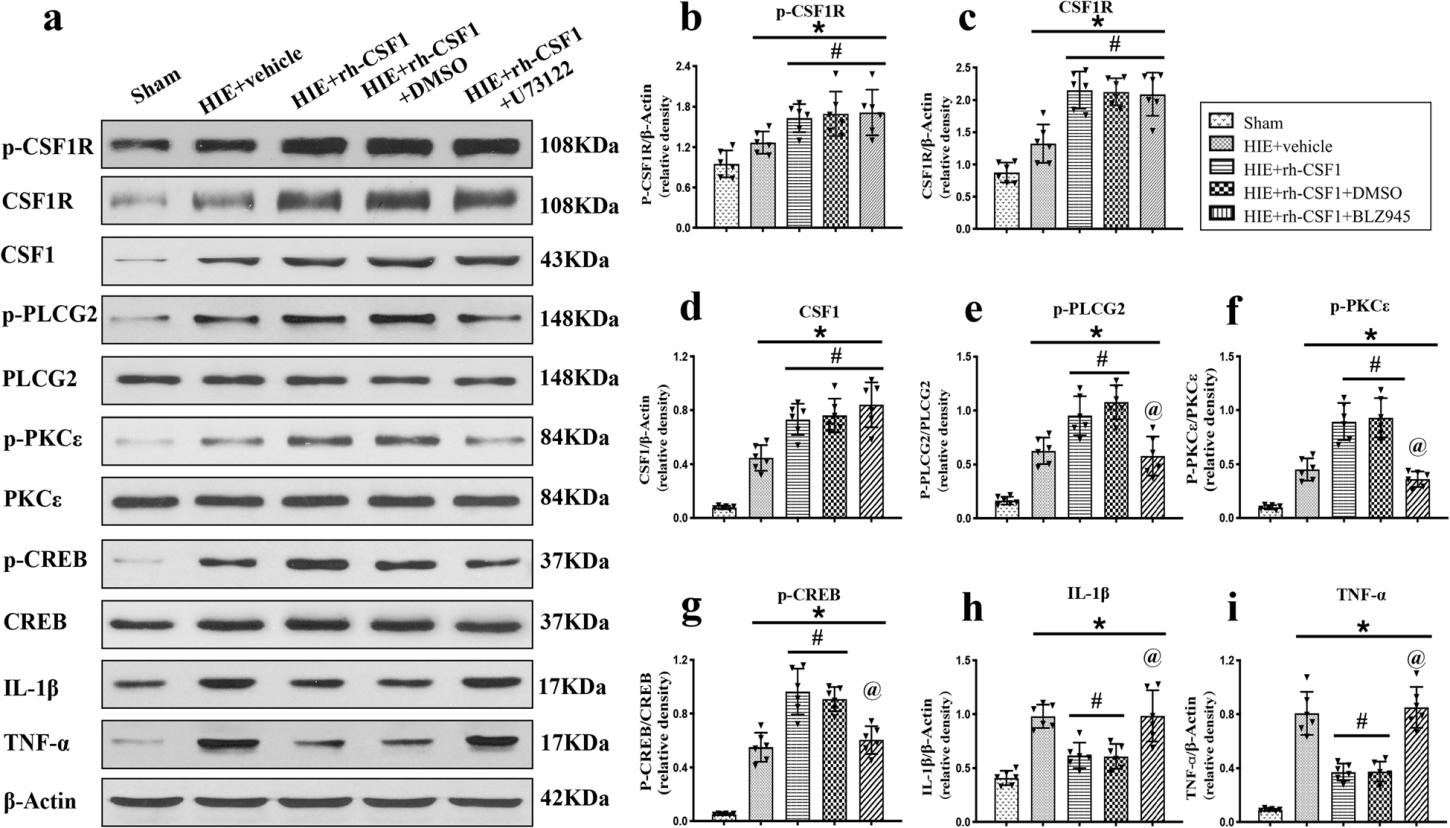
PLCG2 inhibitor U73122 abolished the anti-inflammatory effects of rh-CSF1 via CSF1R/PLCG2/PKCε/CREB signaling pathway at 48 h after HI. **a** Representative western blot bands. **b–i** Densitometric quantification of p-CSF1R, CSF1R, CSF1, p-PLCG2/PLCG2, p-PKCε/PKCε, p-CREB/CREB, IL-1β, and TNF-α in the ipsilateral hemisphere showed that U73122 significantly abolished the effects of rh-CSF1 treatment on protein levels of p-PLCG2, p-PKCε and p-CREB when administered with rh-CSF1. *P < 0.05 vs. sham; ^**#**^*P* < 0.05 vs. HI+ Vehicle; ^**@**^*P* < 0.05 vs. HI+ rh-CSF1 + DMSO; Data are represented as mean ± SD, *n* = 6 in each group

To further clarify the role of downstream proteins, PLCG2 inhibitor was used. The western blot data showed that the PLCG2 inhibitor significantly decreased p-PLCG2 expression, thereby abolishing the effects of rh-CSF1, as evidenced by the attenuated level of p-PKCε and p-CREB, and increased expression levels of IL-1β and TNF-α compared with DMSO group at 48 h after HI (*P* < 0.05, Fig. 9a-i).

The change of pro-inflammatory IL-1β cytokine level in brain tissues (Fig. 8 h and Fig. 9 h) is consistent with the finding of immunofluorescence staining of IL-1β (Fig. 5).

Overall, the intervention of CSF1, CSF1R, and PLCG2 inhibitors did not result in any significant changes in rectal temperature, heart rate, and respiration rate compared to the corresponding treatment control groups at 48 h after HI (data not shown).

## Discussion

This study is the first to explore the therapeutic effect of rh-CSF1 against neuroinflammation and the potential underlying mechanisms after experimentally inducing HIE in rats. Our findings showed that (1) The expression levels of endogenous p-CSF1R, CSF1R, CSF1, p-PLCG2, p-PKCε, and p-CREB were increased after HI in rats, which peaked at 24 h after HI. CSF1 and CSF1R were expressed on microglia at 48 h after HI. (2) Administration of 80 μg/kg rh-CSF1 remarkably reduced the percentage of the infarcted area, brain edema and body weight loss, improved short- and long-term neurological deficits, accompanied by a decrease in neuroinflammation after HI. (3) Rh-CSF1 treatment significantly upregulated the protein levels of p-CSF1R, CSF1R, p-PLCG2, p-PKCε, and p-CREB but downregulated the protein levels of pro-inflammatory cytokines. (4) CSF1R inhibitor, BLZ945, or PLCG2 inhibitor, U73122, abolished the beneficial effects of rh-CSF1 as seen from our neurological tests and neuroinflammatory markers. Taken together, our findings indicated that CSF1R activation with rh-CSF1 ameliorated neuroinflammation, which leads to the improvement of neurobehavioral impairments after HI. This neuroprotection was, at least partly attributed to the activation of microglial PLCG2/PKCε/CREB signaling pathway.

CSF1 is a cytokine of the mononuclear phagocytic system that is upregulated in a variety of CNS diseases, such as AD [46], EAE [47], and neuro HIV [23]. The biological effects of CSF1 are mediated by CSF1R, which is crucial in promoting the migration, proliferation, differentiation, survival, and polarization of macrophage-lineage cells. CSF1R signaling appears to be a necessary condition of growth factor receptor for microglia, which blocks microglial cell death [14]. The expressions of CSF1 and CSF1R were upregulated in HIV CNS disease and autoimmune encephalomyelitis [48, 49]. In this study, our results suggested that the expression levels of CSF1, CSF1R, PLCG2, PKCε, and CREB were increased as early as 6 h and peaked at 24 h after HI. Several damaging factors of brain injury have been proposed to be involved in experimental HIE models, such as neuroinflammation, oxidative stress, neuronal apoptosis, and mitochondria dysfunction [50]. The increased CSF1, CSF1R, PLCG2, PKCε, and CREB expression levels may indicate that they are involved in the endogenous neuroprotective mechanism following HI.

We then tested the effectiveness of rh-CSF1 in the HI model. Based on the time course results and the half-life of rh-CSF1, we treated the pups at 1 h and 24 h after HI. Intranasal administration is a non-invasive approach. The large surface area of the nasal mucosa was conducive to rapid drug absorption and served as a viable and direct method in transporting the drug into the brain [51]. Due to the advantages of intranasal administration, this method was selected to administer rh-CSF1 to neonatal HI rats in our study. Immunofluorescence staining and western blot results verified the expression of exogenous human CSF1 in the rat brain after HI, demonstrating the effectiveness of intranasal administration route of rh-CSF1 into the brain. Our results showed that the intranasal rh-CSF1 at a dose of 80 μg/kg reduced cerebral infarction area, brain edema, negative geotaxis reflex time, and alleviated body weight loss effectively, accompanied by an increase CSF1 positive microglia, and attenuation of neuroinflammation at 48 h after HI. Hippocampal injury can cause long-term cognitive and memory dysfunction after HI [52]. Previous studies reported that the extent of atrophy in the ipsilateral hemisphere and hippocampus was closely associated with short-term sensorimotor dysfunction and long-term neurological functions closely [53]. Our results consistently showed that intranasal administration of rh-CSF1 significantly improved long-term neurological performances of rotarod test, foot-fault test, and morris water maze test, accompanied by the reduction in the volume of brain tissue loss and percentage of brain weight loss in the ipsilateral hemisphere at 4 weeks after HI.

CSF1 is mainly expressed on microglia, neurons, oligodendrocytes, and astrocytes in the CNS [7, 15]. However, CSF1R is primarily localized on microglia [54] and some on neurons in the hippocampus and cortex under physiological conditions [15]. A previous study has been reported that the CSF1R expression was increased in activated microglia after ischemic brain injury in mice [55]. In our study, double immunofluorescence staining indicated that CSF1 and CSF1R were colocalized with microglia at 48 h after HI, which coincided with the previous study. Microglia and/or peripheral infiltration of macrophages play both protective and deleterious roles in the pathology in CNS diseases, which may vary among diseases or may change dynamically as the disease progresses. CSF1 treatment can stimulate the proliferation and activation of microglia, which is protective against neurological dysfunctions and neuroinflammation in a variety of CNS diseases [24, 56–58]. Previous studies have shown that CSF1 improved memory deficits after AD [15], reduced motor behavioral deficits of Parkinson’s disease [59], and ameliorated EAE symptoms [24]. However, microglial depletion by CSF1R inhibitor resulted in dramatically increased the number of neutrophils and augmented the astrocytic production of inflammatory mediators in brain tissues, which eventually exacerbated neurological deficits and brain infarction after ischemic stroke [25, 26]. Furthermore, previous studies have also been reported that CSF1 signaling influenced the phenotype of mononuclear phagocytes, and triggered microglial polarization toward an M2-like phenotype (immunosuppressive), with increased production of anti-inflammatory cytokine IL-10 [60, 61]. All these studies indicated that CSF1 activated proliferation and differentiation of microglia, thus reducing neuroinflammation and playing a neuroprotective role, while the correlation between CSF1 and HIE has not been reported. In the present study, the brain tissue expression levels of CSF1 and CSF1R were increased as well as CSF1/CSF1R positive microglia cells after HI, suggesting an endogenous mechanism of protection. Intranasal administration of rh-CSF1 was able to further enhance the upregulation of CSF/CSF1R signaling. Meanwhile, neuroinflammation is an important component of HIE pathogenesis. We found the significant increases in IL-1β/MPO-positive cells and higher cytokine levels of IL-1β and TNF-α in ipsilateral brain tissues at 48 h after HI. Intranasal rh-CSF1 attenuated the neuroinflammation after HI as well, suggesting that the upregulation of microglia CSF/CSF1R signaling was in favor of suppression of inflammatory responses. Therefore, CSF1R might serve as a novel anti-inflammation target in the setting of HIE.

We further investigated the mechanism underlying the anti-neuroinflammatory effect of CSF1 in HIE. CREB, a transcription factor, responds to a variety of growth factors and inflammatory signals, which mediate the transcription of genes including cAMP-response elements, such as inflammation-related genes of IL-10 and TNF-α [62]. Phosphorylation of CREB protein was activated in the “ischemic penumbra” region in an animal model of transient middle cerebral artery occlusion [63]. Ischemia-resistant dentate granulosa cells had higher levels of CREB phosphorylation than that in ischemia-susceptible CA1 neurons, suggesting CREB phosphorylation as a key factor in achieving ischemic tolerance [63]. The increased CREB phosphorylation has also been shown to be neuroprotective after HI [64]. Interestingly, CSF1 reduced excitotoxic neuronal damage by maintaining the phosphorylation of CREB [15]. In our study, the exogenous rh-CSF1 administration reduced the neuroinflammatory response after HI and promoted the up-regulation of the CREB phosphorylation, which was consistent with previous studies. In addition, BLZ-945, a selective inhibitor of CSF1R, reversed the beneficial effects of CSF1 and significantly downregulated the phosphorylation of CREB. These findings implicated that CSF1 could reduce the inflammatory response after HI by activating CREB signaling. However, the exact mechanism between CSF1/ CSF1R and CREB activation has not yet been elucidated.

The PLCG2 gene provides instructions for making an enzyme, known as phospholipase C gamma 2 (PLCγ2) that catalyzes 1-phosphatidyl-1D-myo-inositol 4,5-bisphosphate to IP3 and DAG using calcium as a cofactor. IP3 and DAG are the second messengers that are important for transmitting signals across cell membranes from growth factor receptors and immune system receptors and are particularly important to immune cells, including B cells, natural killer cells, mast cells, and microglia [29]. Mutations in the gene have been found in spontaneous inflammation, antibody deficiency, neurodegenerative diseases, and dementia [65, 66]. It has been found that CSF1-induced differentiation of human monocytes requires CSF1R activation and downstream PLCG2 phosphorylation [28]. PLCG2 is also involved in regulating microglial function. U73122 is a potent inhibitor of PLCG2, which inhibits the production of IP3 and DAG in human polymorphonuclear neutrophils [67] and affects the cellular viability of microglia [68]. Consistent with these findings, our results showed that intranasal administration of rh-CSF1 increased PLCG2 phosphorylation and reduced neuroinflammation, but these effects were reversed by the co-administration of CSF1R inhibitor U73122. Calcium and DAG can activate the PKC family, including PKCε, which result in the phosphorylation of other molecules and changes in cell activity [69, 70]. PKCε is a protein kinase with a potent anti-inflammatory effect. Waza et al. reported that PKCε regulated the interaction between conexin43 and the ATP sensitive K^+^ channel subunit in cardiomyocyte mitochondria, which prevented hypoxia-induced cell death [71]. Activation of PKCε reduced neuroinflammation [34] and acute ischemic brain injury [72]. Our results showed that rh-CSF1 increased PKCε phosphorylation, which was reversed by PLCG2 and CSF1R inhibitors, suggesting that the activation of CSF1/PLCG2/PKCε signaling pathway contributed to the beneficial effects following neonatal HI. In addition, CSF1R and PLCG2 inhibitors offset the CSF1-induced upregulation of CREB and phosphorylation. These results implied that PLCG2/PKCε was the upstream signaling of CREB in response to CSF1/CSF1R activation. Collectively, these results suggested that rh-CSF1 reduced neuroinflammatory responses, at least in part via the CSF1R/PLCG2/PKCε/CREB signaling pathway.

HIE triggers a robust inflammatory response that exacerbating brain damage post-injury [73]. The acute innate immune response is critical in regulating neonatal HIE injury. Due to gender differences in pro- and anti-inflammatory response, male infants are more susceptible to HI insult and develop severer long-term cognitive deficits than females with comparable brain damage [74]. Microglial activation and accumulation were previously identified as pathological markers for HIE in human infants [75]. However, emerging evidence has shown that microglia play a dual role after brain injury, which can be pro-inflammatory or beneficial depending on its phenotype status [76]. In a rat model of HIE, the microglial depletion aggravated neuronal damage and apoptosis after the HI insult, implicating the early neuroprotection provided by resident microglia [77]. We found that rh-CSF1 promotes the anti-neuroinflammation status of microglia by activation CSF-1R after HI, leading to improved short-term neurological deficits and long-term cognitive function. These results suggest the potential of rh-CSF1 to serve as a new therapeutic approach in HIE patients. The intranasal administration route is invasive and readily clinical translatable. Indeed, recent studies have demonstrated the importance of CSF-1R signaling in microglial and neural development and function in which CSF-1R activations resulted in pleotropic protective effects in multiple neurological diseases [78]. In addition to microglia regulation, rh-CSF-1 may exert directly activating the neuronal CSF-1R and promoting neuronal survival after HI. Neuronal depletion of CSF-1R exacerbated the excitotoxicity injury and subsequent neurodegeneration [15]. Systemic CSF-1 administration increased the neuronal expression of p-CREB, an essential survival signaling, with the resultant improvement of cognitive function in a mouse model of AD [15]. Although our focus is neuroinflammation in the current study, the neuronal effects of rh-CSF1 need to be further explored in the setting of experimental HIE.

There are several limitations to this study. First, previous studies showed that the CSF1 was also expressed in astrocytes and neurons. The anti-apoptotic effects and blood-brain barrier protection of CSF1 after HI requires further investigation. Second, CSF1 could also induce autophagy to regulate human monocyte differentiation through the CAMKK2/PRKAA1/ULK1 signaling pathway [28]. However, we could not exclude the contribution of other signaling pathways to the anti-inflammatory effects of CSF1 in the HI model. Lastly, we did not test the effects of rh-CSF1 on female rat pups. There is increasing evidence suggests that HIE is a sexually dimorphic disease [74]. Future studies are warranted to validate the other neuroprotective functions and potential mechanisms as well as gender differences associated with CSF1 after HI.

## Conclusions

In conclusion, the intranasal administration of rh-CSF1 reduced the percentage of infarcted areas, brain edema, and improved neurobehavioral deficits, by attenuating neuroinflammation after HI in rats. The anti-inflammatory effects of CSF1 were, at least in part, mediated through the CSF1R/PLCG2/PKCε/CREB signaling pathway. Thus, rh-CSF1 may serve as a novel therapeutic approach to ameliorate brain injury in patients with HIE.

## Supplementary information

**Additional file 1: Figure S1.** Recombinant human CSF1 (rh-CSF1) attenuates neuroinflammation via the CSF1R/PLCG2/PKCε/CREB signaling pathway in a rat model of neonatal HIE.

## Data Availability

The data support the findings of this study and are available from the corresponding author upon reasonable request.

## Abbreviations

AD: Alzheimer's disease
CNS: Central nervous system
CREB: Cyclic adenosine monophosphate response element-binding
CSF1: Colony-stimulating factor 1
CSF1R: Colony-stimulating factor 1 receptor
DAG: Diacylglycerol
EAE: Experimental autoimmune encephalomyelitis
HIE: Hypoxic-ischemic encephalopathy
IACUC: Institutional Animal Care and Use Committee
IP3: Inositol 1,4,5 trisphosphate
PKCε: Protein kinase C epsilon
PLCG2: Phospholipase C-gamma 2
TTC: Triphenyltetrazolium chloride
