# Supplementary figures and images for: Rh-CSF1 attenuates neuroinflammation via the CSF1R/PLCG2/PKCε pathway in a rat model of neonatal HIE
Source: J Neuroinflammation. 2020 Jun 10;17:182. doi: 10.1186/s12974-020-01862-w (PMC7285566; doi:10.1186/s12974-020-01862-w)

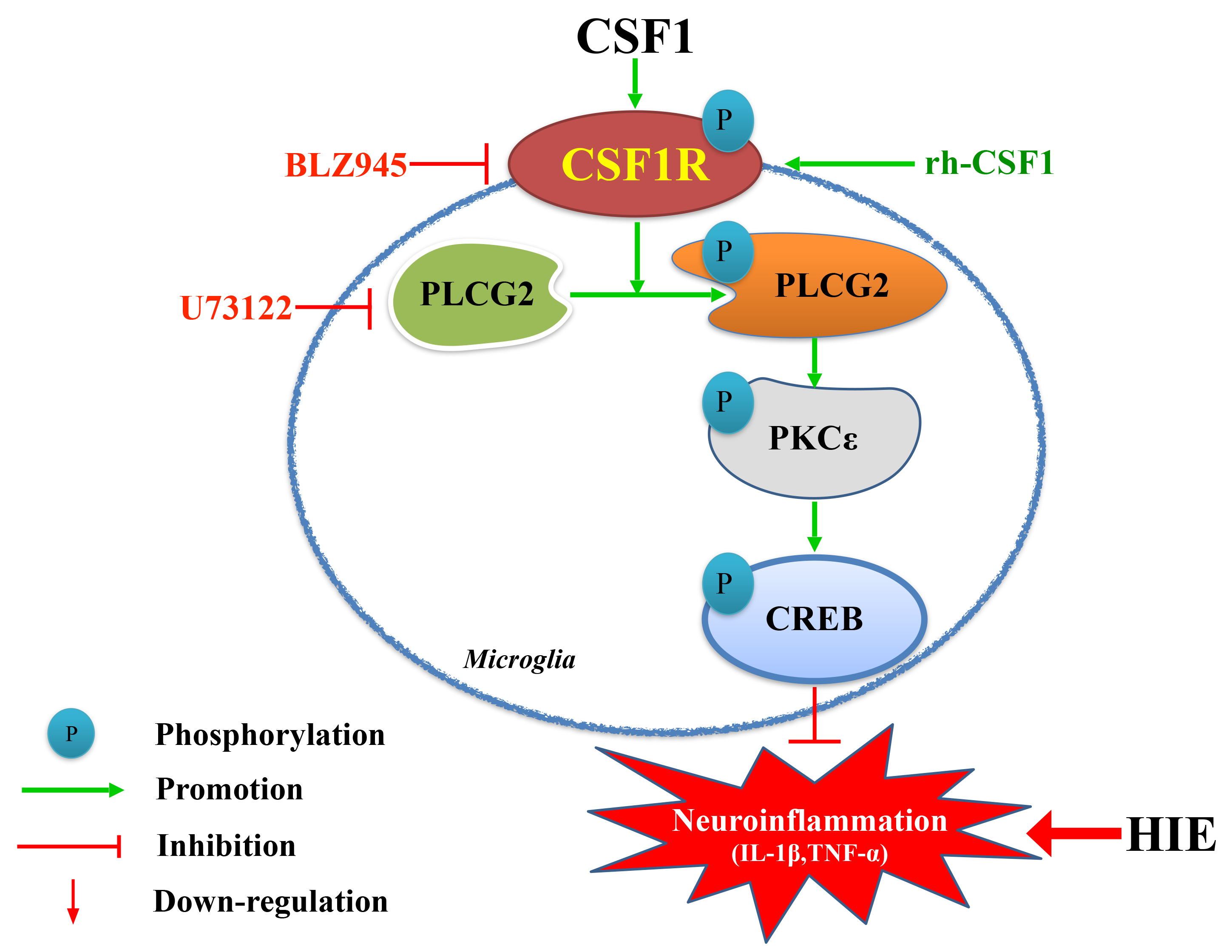

Supplement: Supplementary file 1 — Additional file 1: Figure S1. Recombinant human CSF1 (rh-CSF1) attenuates neuroinflammation via the CSF1R/PLCG2/PKCε/CREB signaling pathway in a rat model of neonatal HIE. [file 12974_2020_1862_MOESM1_ESM.tif]
